# Novel gait training alters functional brain connectivity during walking in chronic stroke patients: a randomized controlled pilot trial

**DOI:** 10.1186/s12984-019-0503-2

**Published:** 2019-02-28

**Authors:** I-Hsuan Chen, Yea-Ru Yang, Chia-Feng Lu, Ray-Yau Wang

**Affiliations:** 1 Department of Physical Therapy, Fooyin University, Kaohsiung, Taiwan; 2 Department of Physical Therapy and Assistive Technology, National Yang-Ming University, 155, Sec 2, Li Nong St., Shih-Pai, Taipei, 112 Taiwan; 3 Department of Biomedical Imaging and Radiological Sciences, National Yang-Ming University, Taipei, Taiwan

**Keywords:** Stroke, EEG, EMG, Gait, Brain connectivity, Turning

## Abstract

**Background:**

A recent study has demonstrated that a turning-based treadmill program yields greater improvements in gait speed and temporal symmetry than regular treadmill training in chronic stroke patients. However, it remains unknown how this novel and challenging gait training shapes the cortico-cortical network and cortico-spinal network during walking in chronic stroke patients. The purpose of this study was to examine how a novel type of gait training, which is an unfamiliar but effective task for people with chronic stroke, enhances brain reorganization.

**Methods:**

Subjects in the experimental and control groups received 30 min of turning-based treadmill training and regular treadmill training, respectively. Cortico-cortical connectivity and cortico-muscular connectivity during walking and gait performance were assessed before and after completing the 12-session training.

**Results:**

Eighteen subjects (*n* = 9 per group) with a mean age of 52.5 ± 9.7 years and an overground walking speed of 0.61 ± 0.26 m/s consented and participated in this study. There were significant group by time interactions for gait speed, temporal gait symmetry, and cortico-cortical connectivity as well as cortico-muscular connectivity in walk-related frequency (24–40 Hz) over the frontal-central-parietal areas. Compared with the regular treadmill training, the turning-based treadmill training resulted in greater improvements in these measures. Moreover, the increases in cortico-cortical connectivity and cortico-muscular connectivity while walking were associated with improvements in temporal gait symmetry.

**Conclusions:**

Our findings suggest this novel turning-based treadmill training is effective for enhancing brain functional reorganization underlying cortico-cortical and corticomuscular mechanisms and thus may result in gait improvement in people with chronic stroke.

**Trial registration:**

ACTRN12617000190303. Registered 3 February 2017, retrospectively registered.

## Introduction

A recent study suggested that chronic stroke patients maintain the capacity to increase synchronization of neural activity between different brain regions as measured by EEG connectivity. These changes of functional connectivity in the motor cortex through neurofeedback correlate with improvements in motor performance [1]. Previously, we demonstrated that a novel specific training, the turning-based treadmill program, yielded greater improvements in gait speed and temporal symmetry than regular treadmill training for people with chronic stroke [2]. We presumed the turning-based treadmill training, which is a challenging and unfamiliar training task for chronic stroke patients, may facilitate brain reorganization and behavioral recovery [3]. Thus, we sought to understand how such novel gait training promotes brain reorganization in this study.

An EEG-based method has the advantage of real-time recording during walking due to the relative ease of data acquisition. As indicated by the authors of the first study to use an EEG signal recorded during walking, the power increases within numerous frequency bands (3–150 Hz) in the sensorimotor cortex and is more pronounced during the end of the stance phase of walking [4]. Source localization EEG analysis revealed the importance of the primary somatosensory, somatosensory association, primary motor and cingulate cortex in gait control [5]. Focal lesions due to stroke may not only affect the functional connectivity of cortical areas [6] but also impede the neural transmission of descending motor pathways [7]. Based on spectral analysis, the direct relationship of cortical activities with peripheral movements is still unknown. Accordingly, an analysis of EEG-EMG coherence recorded during treadmill walking was done by Petersen et al. [8], who demonstrated that cortical activity in the primary motor cortex within the gamma band (24–40 Hz) was transmitted via the corticospinal tract to the leg muscles during the swing phase of walking. In addition, a recent study confirmed the strong correlation between kinematic errors of the lower extremities and fronto-centroparietal connectivity during gait training and post-training in healthy subjects [9]. However, it remains unknown how novel and challenging gait training shapes the cortico-cortical network and cortico-spinal network during walking in individuals with chronic stroke. Therefore, the aims of the current study were to explore the effects of the turning-based treadmill training, a novel gait training program, on cortico-cortical connectivity and corticomuscular connectivity and to investigate the relationship between connectivity changes and gait performance in chronic stroke patients.

## Methods

### Participants

Participants with chronic stroke were recruited from medical centers and the surrounding community. Stroke diagnosis, age, gender, stroke type, lesion side, and duration of hemiparesis were obtained from patient interviews and medical charts. To be included in the study, participants with stroke had to satisfy the following criteria: (a) first stroke with unilateral motor deficits at least 6 months prior, (b) ability to walk independently for at least 6 m with or without the use of stick, quad stick or AFO (to ensure they were able to complete the walk test used in this study), (c) a Brunnstrom stage greater than 3 for the affected lower extremity, and (d) a score of ≥24 on the mini-mental state examination (MMSE). Exclusion criteria were (a) unstable medical conditions (e.g., deep vein thrombosis, aspiration pneumonia, or superimposed sepsis) and (b) history of other diseases known to interfere with participation in such a study (e.g., heart failure, hemi-neglect, or diabetic neuropathy).

### Experimental design

The study was a single-blind, parallel randomized, controlled trial. A research assistant enrolled the participants at the medical centers. An individual unassociated with any other study procedure generated the random allocation sequence and selected one of a set of sealed envelopes to assign participants to either the experimental or control group (block randomization; allocation ratio = 1) before the intervention began. Participants received 30 min of turning-based treadmill training (experimental group) or regular treadmill training (control group), followed by a 10-min ambulation training program, for 12 sessions over a 4-week period. All training procedures were implemented under a physical therapist’s supervision. All outcomes were measured the day before intervention (pre) and the day after completing the intervention (post) by a physical therapist blinded to the group assignment. The study outcomes (see below) included neurophysiological measures during treadmill walking and walking performance on the ground. Participants provided written informed consent for all study procedures, which were approved by the institutional review board of Taipei City Hospital. This trial was registered at https://www.anzctr.org.au/Trial/Registration/TrialReview.aspx?id=372132 (ACTRN12617000190303).

### Interventions

#### Turning-based treadmill training

A rotational treadmill was designed to provide turning-based treadmill training [2]. This treadmill is similar to a regular treadmill except for its circular running motor belt (0.8-m radius), which forces patients to continually turn rather than walk straight. Participants walked on the perimeter of the circular belt as it rotated either clockwise or counterclockwise. Participants were trained in both directions, with the affected leg as the inner limb for 15 min and then as the outer limb for 15 min with a 5-min break in between. The order of walking with the affected limb as the inner or outer leg was alternated in each consecutive session. All participants wore an unweighted safety harness to prevent falls. Participants were also allowed to place their hands on the handrail for balance support but were instructed to refrain from holding the handrail. As progressively faster speeds are needed to continue challenging the locomotor abilities of individuals with hemiparesis [10], the treadmill speed, which began at each individual’s comfortable turning speed on level ground, was increased by increments of 0.05 m/s every 5 min as tolerated. After completing the turning-based treadmill training, a 10-min ambulation training followed [2].

#### Regular treadmill training

The control group received training on a standard treadmill (Biodex, Shirley, NY). Other than the type of treadmill, the training protocol was the same as that described for the experimental group. Training speed was initially set at the individual’s comfortable walking speed on level ground and increased by increments of 0.05 m/s every 5 min as tolerated. Participants were trained in two 15-min phases, followed by a 10-min ambulation training as in the experimental group.

### Outcome measures

#### Functional brain connectivity during walking

The motor system consists of well-tuned interactions of excitatory and inhibitory mechanisms in the cortical and subcortical areas. Many researchers have used coherence analysis, a method of power spectral cross correlations between spatially separate neural systems, to reflect the functional communication between two systems, i.e., coupling between two brain regions (EEG-EEG coherence) [6], or between cortical commands and consequent muscle activation (EEG-EMG coherence) [7]. However, the conventional coherence methods show linear dependency and may be insufficient to study the nonlinear signals in biological and physical systems, such as data pertaining EEG and EMG [11], especially if the signals are contaminated by noise or the oscillatory frequency band is not carefully defined [12, 13].

In the present study, the time-frequency cross mutual information (TFCMI) method was used to solve the problems mentioned above, to calculate the mutual information between two temporal power sequences within a task-specific band between two neural systems [14, 15], and thus to examine the treatment-induced brain reorganization during walking. To obtain consistent information, in both the experimental and control groups, subjects walked on the regular treadmill for 5 min while the neurophysiological measures and the functional connectivity of the signals from different scalp locations (EEG-EEG connectivity) and the brain and muscle (EEG-EMG connectivity) were assessed .

EEG, EMG, and footswitch signals during treadmill walking were simultaneously recorded from Ag/AgCl electrodes using a 40-channel QuickAmp amplifier (32 EEG channels, 4 bipolar channels for EMG and 4 auxiliary channels for footswitch) and Brain Vision Recorder software (Brain Products, Gilching, Germany).

Using the international 10–20 system, 32 EEG electrodes located over the frontal-parietal areas were used (F5, F3, F1, Fz, F2, F4, F6, FC5, FC3, FC1, FCZ, FC2, FC4, FC6, C5, C3, C1, CZ, C2, C4, C6, CP5, CP3, CP1, CPZ, CP2, CP4, CP6, P1, Pz, P2, POz). Electrodes were recorded against an average reference calculated by the amplifier hardware. Horizontal and vertical electrooculography (HEOG and VEOG) signals were also recorded. EEG and EOG signals were amplified with a bandpass of 0.16–50 Hz, recorded in DC mode and sampled at 1000 Hz. Electrode impedances were kept below 5 kΩ [16].

For recording EMG, the skin was shaved and cleaned with alcohol swabs to reduce impedance before applying 11 mm Ag-AgCl surface electrodes (Brain Products, Gilching, Germany). Surface electrodes were placed on the motor point of the tibialis anterior (TA) of the affected leg, which is located at 1/3 on the line between the tip of the fibular and the medial malleolus [17]. Electrode impedances were kept below 5 kΩ.

Four footswitches were placed respectively under the heel and the big toe of each leg and taped to the sole of the foot using medical tape to measure the “heel strike” and “toe-off” events of the gait cycle. Signals from footswitches were recorded using the QuickAmp amplifier and Brain Vision Recorder software to synchronize signals from footswitches with those of the EEG and EMG. Significant coupling between EEG and EMG signals using the conventional EEG-EMG coherence method was found approximately 24–40 Hz during the swing phase of walking in healthy human subjects [8]. Therefore, the target gait event in this study was focused on the swing phase. The swing phase was defined as the time interval between toe-off and the subsequent heel strike of the same foot.

EEG-EEG connectivity and EEG-EMG connectivity were calculated using the TFCMI method. The data recorded within the target gait event was off-line processed as follows. The independent component analysis (ICA) algorithm was first applied to decompose EEG recordings into 15 independent components. Among these independent components, the signals that were highly correlated to the VEOG or HEOG (with correlation coefficients higher than 0.6) were rejected from the reconstruction to eliminate the potential contamination from eye movement during recording. The EEG/EMG recordings were then segmented into epochs measuring from 200 sample points before the onset of the “toe-off” to 300 sample points after the onset of the “toe-off”. The epochs with a signal fluctuation more than a standard deviation from the mean signal value were then discarded to remove potential motion artifact and ensure signal stability. The stable EEG signal over time within the same patient was obtained through ICA-based artifact rejection. The Butterworth zero-phase filters with pass band from 1 to 50 Hz were applied. Each window of filtered EEG/EMG data across channels was transformed into the time–frequency domain using the Morlet wavelet transformation to obtain temporal spectral (power) map [18]. The corresponding Morlet wavelet transformation was given by time–frequency maps encompassing the alpha (8–12 Hz) and gamma (24–40 Hz) activities that were created separately within each window. The power across the selected frequency bands in each channel was averaged to produce power curves of the target bands, i.e., alpha and gamma bands in this study. The temporal series of the averaged power signals of the target bands in each channel were used to compute the cross mutual information between any two channels in each window. A full mathematical description of TFCMI has been published previously [14]. The TFCMI value, which is an index of functional connectivity that estimates the relationship between two channels on their averaged power changes over the target band, was calculated based on the entropy and joint entropy. The TFCMI between the i^th^ and j^th^ channels were calculated as follows:

$$ \mathrm{TFCMI}\left({F}_i,{F}_j\right)=H\left({F}_i\right)+H\left({F}_j\right)-\left({F}_i,{F}_j\right)={\sum}_{b=1}^{50}p\left({F}_{i,b},{F}_{j,b}\right)\ln \frac{p\left({F}_{i,b},{F}_{j,b}\right)}{p\left({F}_{i,b}\right)p\left({F}_{j,b}\right)} $$

H: the entropy; F: the averaged power signals at the channel; P: the probability density function (pdf); b: the index of sampling bins used to construct the approximated pdf.

The cortical maps of EEG-EMG connectivity were presented in individual channels (32 channels). Considering pairwise connectivity, a 32-channel network for the EEG-EEG connectivity may produce (32 × 32–32)/2 possible functional connections, which may be convoluted when visualized. Therefore, the 32 channels were grouped into 13 regions to better visualize the EEG-EEG connection between cortical regions (Table 1).

**Table 1:** The divided cortical regions and their abbreviations

Abbreviation	Cortical location	Channels
IF	Ipsilesional frontal area	F4, F6
MF	Middle frontal area	F1, Fz, F2
CF	Contralesional frontal area	F5, F3
IFC	Ipsilesional frontal-central area	FC4, FC6
MFC	Middle frontal-central area	FC1, FCz, FC2
CFC	Contralesional frontal-central area	FC5, FC3
IC	Ipsilesional central area	C4, C6
MC	Middle central area	C1, Cz, C2
CC	Contralesional central area	C5, C3
ICP	Ipsilesional central-parietal area	CP4, CP6
MCP	Middle central-parietal area	CP1, CPz, CP2
CCP	Contralesional central-parietal area	CP5, CP3
MP	Middle parietal area	P1, Pz, P2, POz

#### Gait performance

The gait speed and symmetry measure independent features. Gait speed provides the overall gait performance and may differentiate levels of disability, while the temporal gait symmetry indicates differences of single support time between two limbs due to the difficulty of bearing weight on the affected leg during the stance phase or in advancing the affected leg during the swing phase [19, 20]. The speed and temporal asymmetry ratio at a comfortable walking speed were obtained from the GAITRite system (CIR system, Inc., Havertown, PS); these two gait parameters were selected based on the results of our previous study [2]. The concurrent validity [21] and reliability [22] of the GAITRite system for stroke subjects have been established. The GAITRite walkway was 4.75 m long and 0.9 m wide, and the pressure-sensitive area was 4.30 m long and 0.61 m wide. The contact time and location of each footfall were recorded and the gait parameters were calculated on a laptop with application software. Participants began their trials 1.5 m before the mat and continued walking for 1.5 m beyond the end of the mat to eliminate the effect of acceleration and deceleration [22]. Participants were allowed to use their customary ankle-foot orthosis or assistive devices [22]. The assistive device and/or ankle foot orthosis used at the preintervention assessment were also used at the postintervention assessment. Fragmented steps and unrelated marks from assistive devices were removed automatically by the GAITRite software or manually by the investigators [22]. The average of three trials was used for data analysis [22]. The temporal asymmetry ratio was calculated using the following formulas [2]:

$$ \mathrm{Temporal}\ \mathrm{asymmetry}\ \mathrm{ratio}=\left|1-\frac{\mathrm{single}\ \mathrm{support}\ \mathrm{time}\ \left(\mathrm{affected}\right)}{\mathrm{single}\ \mathrm{support}\ \mathrm{time}\ \left(\mathrm{unaffected}\right)}\right| $$

### Statistical analysis

All analyses were performed using the SPSS 17.0 statistical software. Descriptive statistics were generated, and distributions of the variables were expressed as the mean ± standard deviation. Intergroup differences among baseline characteristics were evaluated using an independent t test or χ2 analysis. Two-way analysis of variance with repeated measures was used to determine the effects of intervention on each dependent variable. Model effects were group (experimental, control), time (pre, post), and their interactions. Post hoc independent t tests between groups were used to examine significant models. The association between neurophysiological measures during treadmill walking with significant intergroup differences and training-related changes in gait performance was performed using a Pearson correlation test. Statistical significance was set at .05.

## Results

Nine participants in the experimental group and 9 participants in the control group completed the study protocol and had sufficient successful trials of functional and neurophysiological data. No adverse effects were reported or noted. The characteristics and clinical information of participants are shown in Table 2. There were no significant differences in baseline demographic or clinical features between the groups. Similarly, there were no significant group differences in any of the preintervention outcome measures.

**Table 2:** Baseline demographic and clinical characteristics of participants

Parameters	Control group (*n* = 9)	Experimental group (*n* = 9)	*P*
Age, y	50.33 ± 10.95	54.67 ± 8.32	0.359
Gender, male/female	8/1	9/0	1.000
Body weight, kg	68.33 ± 9.77	66.33 ± 8.44	0.648
Height, cm	168.0 ± 6.42	168.89 ± 4.34	0.735
Side of hemiparesis, right/left	3/6	3/6	1.000
Type of stroke, hemorrhagic/ischemic	6/3	3/6	0.347
Time poststroke, y	3.08 ± 2.17	2.86 ± 2.40	0.839
Brunnstrom stage of lower extremity	4.5 ± 0.8	4.3 ± 0.7	0.644
Walking aids used, yes/no	5/4	3/6	0.637
Rehabilitation ongoing, yes/no	8/1	8/1	1.000

Values are the mean ± standard deviation or frequency

### Functional brain connectivity during walking

**Fig. 1:**
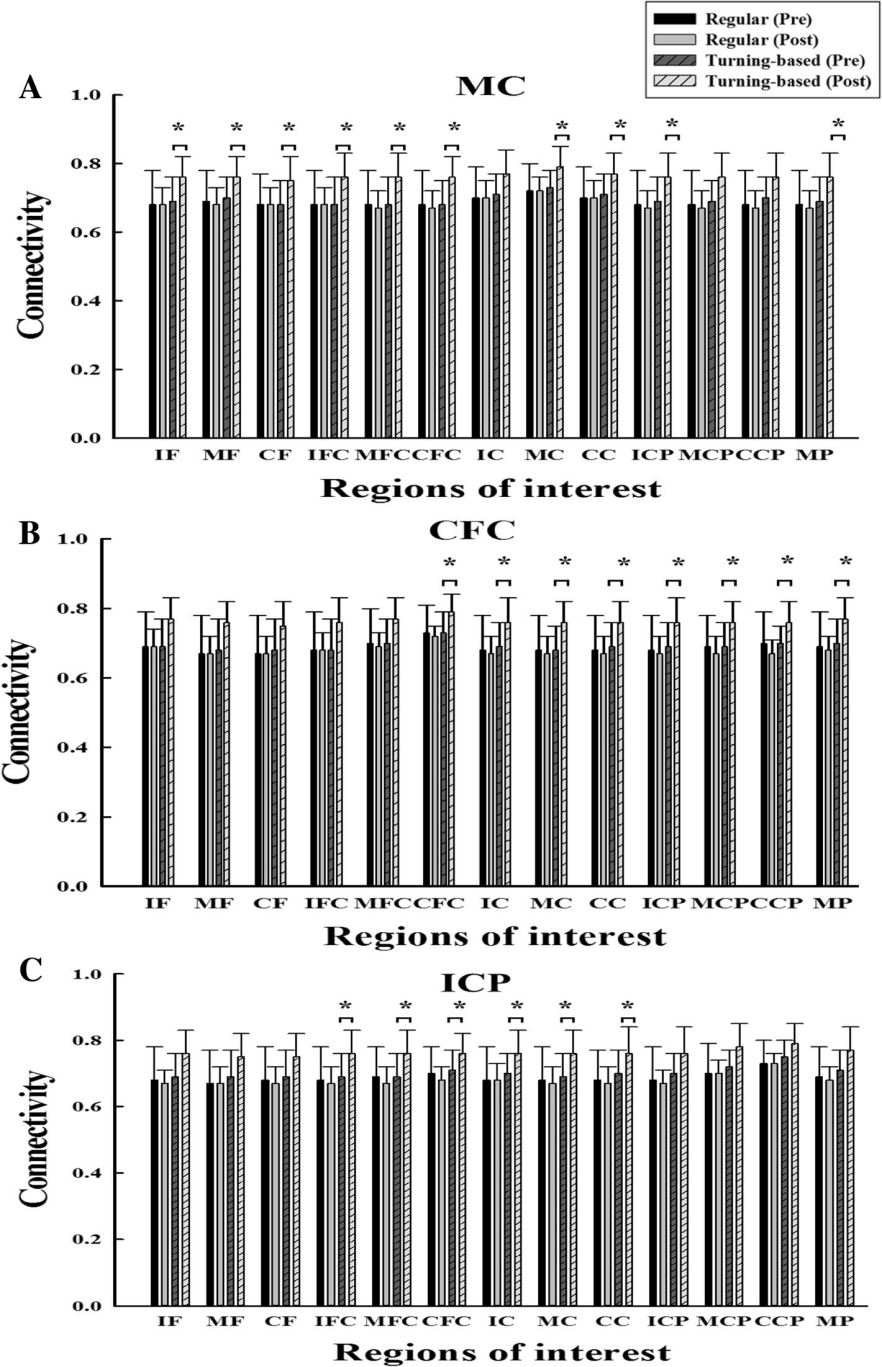
The mean and standard variations of the EEG-EEG connectivity values in the gamma band are shown for the (**a**) MC-13 regions, (**b**) CFC-13 regions, and (**c**) IPC-13 regions. * denotes a significance level < 0.05 for intergroup comparisons (control vs. experimental)

**Fig. 2:**
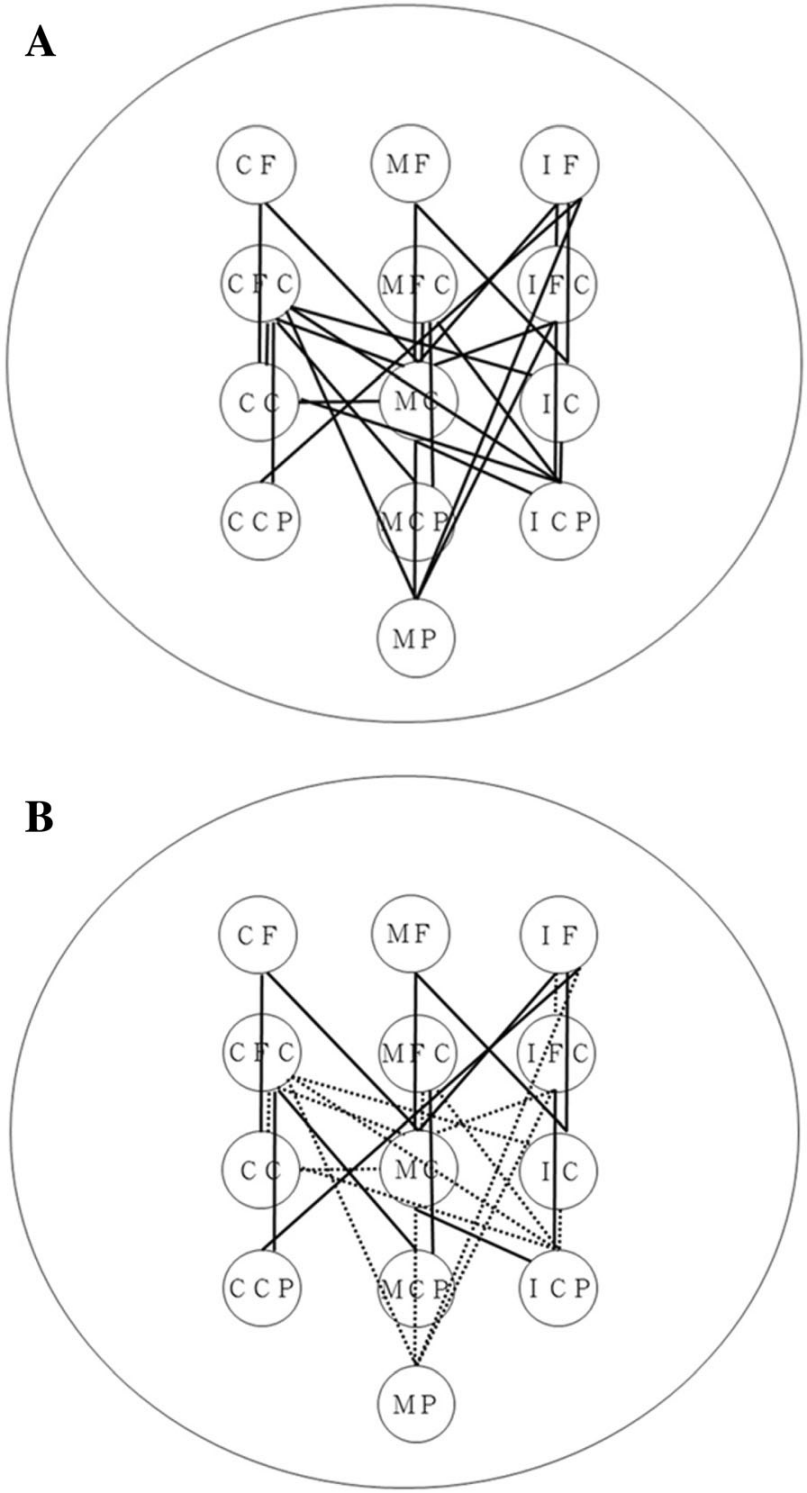
(**a**) Functional EEG-EEG connectivity in the gamma band with significant intergroup differences. The signals were recorded during regular treadmill walking. The lines represent the between-region connectivity. (**b**) Changes in functional EEG-EEG connectivity in the gamma band correlate with the recovery of temporal gait asymmetry. The solid lines represent the changes in the temporal asymmetry ratio that were negatively correlated with the changes in the EEG-EEG pairs. The dotted lines represent the changes in the temporal asymmetry ratio that were not correlated with the changes in the EEG-EEG pairs. For better visualization and interpretation, the left represents the contralesional hemisphere, and the right represents the ipsilesional hemisphere. The abbreviations of the cortical areas are listed in Table 1

The results of TFCMI analysis in EEG-EEG connectivity are shown in Figs. 1 and 2. Significant group by time interactions were noted in the gamma band. Post hoc testing showed that the experimental group demonstrated significant increases in the EEG-EEG connectivity in multiple areas compared with those of the control group. The experimental group demonstrated significant pre- and postintervention differences in EEG-EEG connectivity, but the control group did not show significant pre- and postintervention differences. The connectivity increases are primarily distributed in the middle central area (MC-IF, MC-MF, MC-CF, MC-IFC, MC-MFC, MC-CFC, MC-MC, MC-CC, MC-ICP, MC-MP) (Fig. 1a), secondarily in the contralesional frontocentral regions (CFC-CFC, CFC-IC, CFC-MC CFC-CC, CFC-ICP, CFC-MCP, CFC-CCP, CFC-MP) (Fig. 1b), and thirdly in the ipsilesional centroparietal regions (ICP-IFC, ICP-MFC, ICP-CFC, ICP-IC, ICP-MC, ICP-CC) (Fig. 1c). The increase in the interhemisphric connectivity included 4 pairs, IF-CCP, CFC-IC, CFC-ICP, and CC-ICP. However, no significant interactions were observed in the alpha band. Figure 2a depicts the areas (total 28 pairs) exhibiting increases in connectivity.

**Fig. 3:**
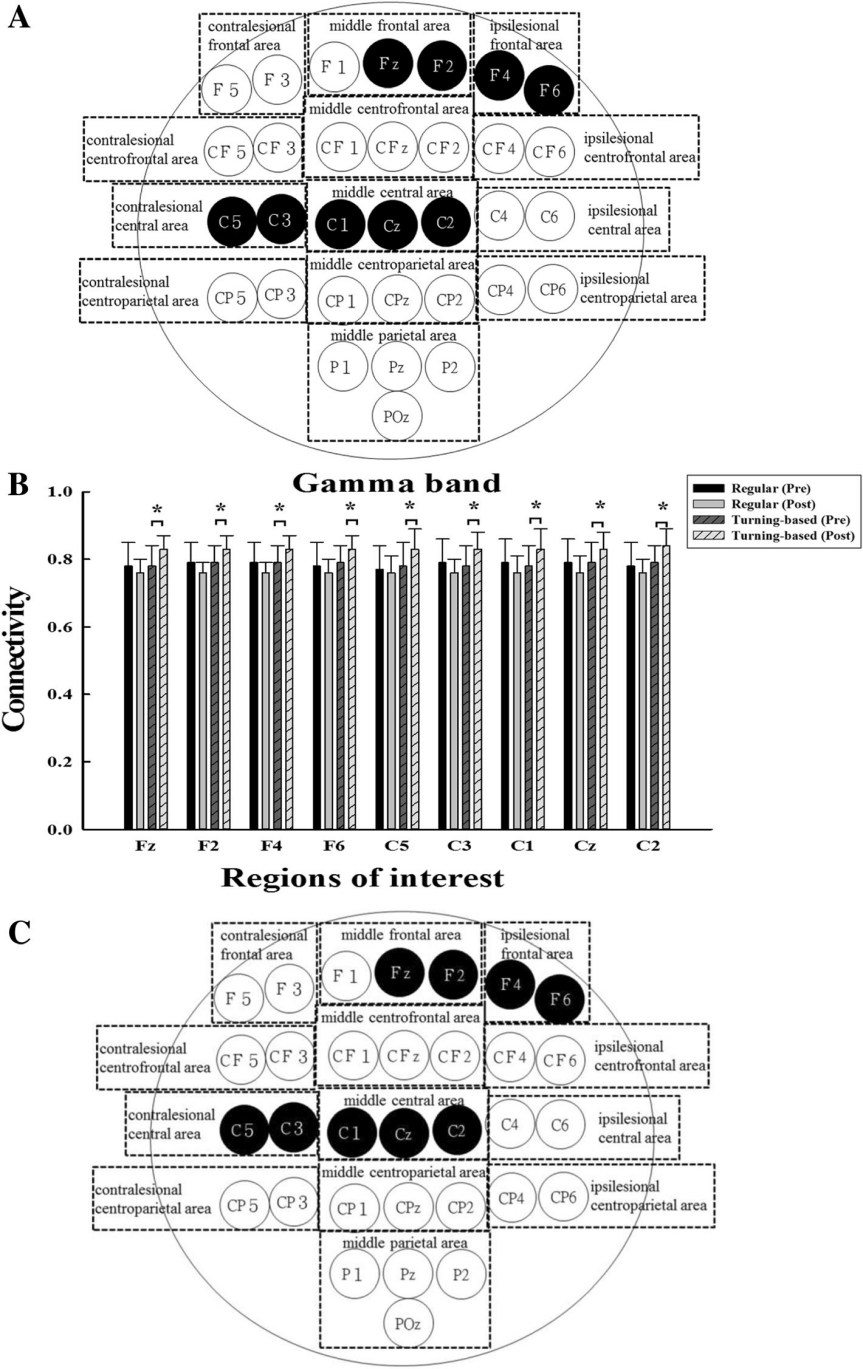
(**a**) Functional EEG-EMG connectivity in the gamma band. The signals were recorded during regular treadmill walking. The black circles represent the corticomuscular connectivity with significant intergroup difference. (**b**) The means and standard deviations of the EEG-EMG connectivity values are shown for the regions with significant intergroup differences. * denotes a significance level < 0.05 for intergroup comparisons (control vs. experimental). (**c**) Changes in the functional EEG-EMG connectivity in the gamma band correlate with the recovery of temporal gait asymmetry. The black circles represent the changes in the EEG-EMG pairs with significant intergroup differences that were negative correlated with the changes in the temporal asymmetry ratio. For better visualization and interpretation, the left represents the contralesional hemisphere, and the right represents the ipsilesional hemisphere

**Fig. 4:**
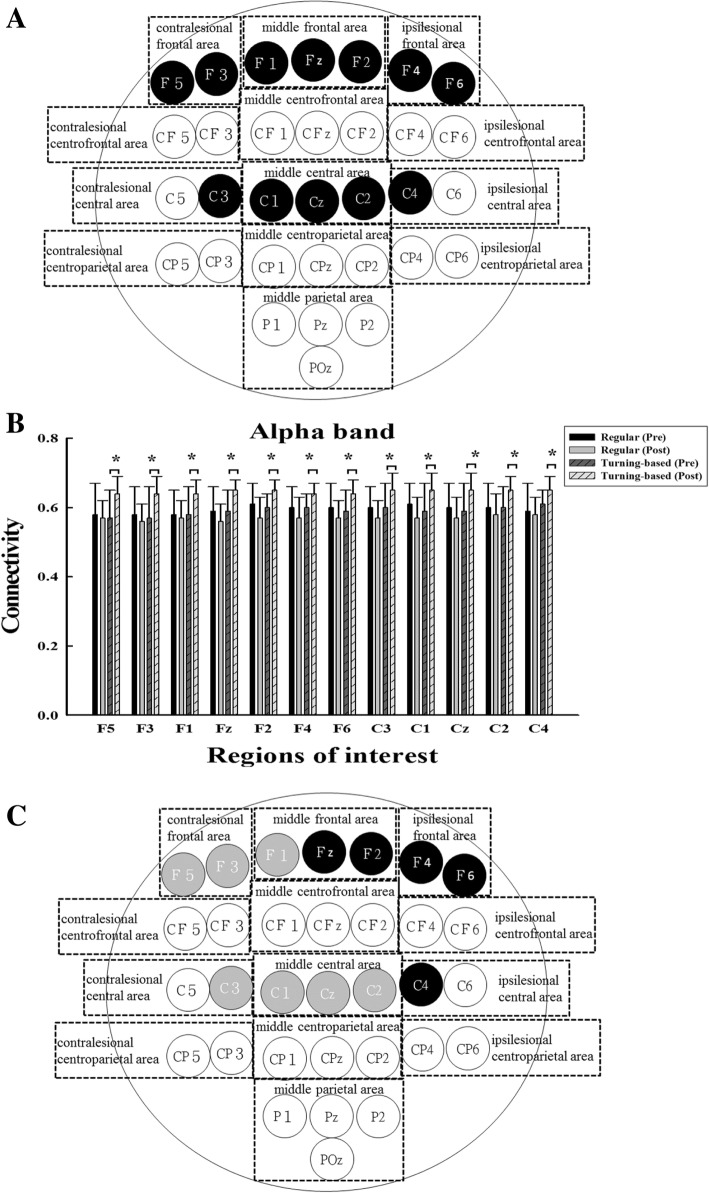
(**a**) Functional EEG-EMG connectivity in the alpha band. The signals were recorded during regular treadmill walking. The black circles represent the corticomuscular connectivity with significant intergroup differences. (**b**) The means and standard deviations of the EEG-EMG connectivity values are shown for the regions with significant intergroup differences. * denotes a significance level < 0.05 for intergroup comparisons (control vs. experimental). (**c**) Changes in the functional EEG-EMG connectivity in the alpha band correlate with the recovery of temporal gait asymmetry. The black circles represent the changes in the EEG-EMG pairs with significant intergroup differences that negatively correlate to the changes in the temporal asymmetry ratio. The gray circles represent the changes in the EEG-EMG pairs with significant intergroup differences that do not correlate to the changes in the temporal asymmetry ratio. For better visualization and interpretation, the left represents the contralesional hemisphere, and the right represents the ipsilesional hemisphere

The results of TFCMI analysis in EEG-EMG connectivity are shown in Figs. 3 and 4. In the gamma band, significant interactions between group and time were observed for EEG-EMG connectivity over the frontal and central areas. Post hoc analysis revealed that the experimental group demonstrated significant increases in EEG-EMG connectivity over middle frontal area (Fz, F2), ipsilesional frontal area (F4, F6), contralesional central area (C3, C5), and middle central area (C1, Cz, C2) after training compared to the control group (Fig. 3a and b). The experimental group demonstrated significant pre- and postintervention differences in the EEG-EMG connectivity, but the control group did not show significant pre- and postintervention differences.

For the alpha band, significant interactions between group and time were observed over the frontal and central areas. Post hoc testing indicated that the experimental group demonstrated significant increases in the EEG-EMG connectivity over the contralesional frontal area (F5, F3), middle frontal area (F1, Fz, F2), ipsilesional frontal area (F4, F6), contralesional central area (C3), middle central area (C1, Cz, C2) and ipsilesional central area (C4) compared to the control group (Fig. 4a and b). The experimental group demonstrated significant pre- and postintervention differences for almost all EEG-EMG connectivity, except for F3, C3, and Cz, but the control group did not show significant pre- and postintervention differences.

### Gait performance

Group by time interactions (*p* < 0.040) were noted for both walking speed and asymmetry ratio (Table 3). Compared to the control group, the experimental group demonstrated greater improvements in walking speed (*p* = 0.037) and temporal asymmetry ratio (*p* = 0.026). For the within group comparisons, the experimental group improved significantly from pre- to postintervention for both gait speed (*p* = 0.011) and asymmetry ratio (*p* = 0.006), but the control group did not show significant differences in gait speed and asymmetry ratio. The individual gait details assessed before and after intervention are presented in the Table 4. All participants, except for #2 in the control group (Table 4), demonstrated the “temporal asymmetry” at the beginning (normative range: < 0.1) [23].

**Table 3:** Gait performance

	Control group (n = 9)		Experimental group (n = 9)		Time Effect, *P*	Time × Group, *P*
Measures	Pre	Post	Pre	Post		
Speed (m/s)	0.64 ± 0.30	0.66 ± 0.31	0.58 ± 0.23	0.71 ± 0.31*	0.01	0.037
Temporal asymmetry ratio	0.29 ± 0.16	0.31 ± 0.15	0.30 ± 0.12	0.24 ± 0.08*	0.138	0.026

Values are the mean ± standard deviation

*Significance level < 0.05 for intergroup comparisons (control vs. experimental)

**Table 4:** Individual data of gait performance

		Speed (m/s)		Temporal asymmetry ratio	
	Subject number	Pre	Post	Pre	Post
Control group	1	1.00	1.02	0.27	0.28
	2	0.82	0.75	0.09	0.1
	3	0.78	0.98	0.12	0.11
	4	0.83	0.73	0.40	0.35
	5	0.21	0.29	0.57	0.46
	6	0.26	0.19	0.24	0.42
	7	0.59	0.67	0.26	0.22
	8	0.88	0.93	0.21	0.30
	9	0.38	0.36	0.48	0.52
	Mean (SD)	0.64 (0.30)	0.66 (0.31)	0.29 (0.16)	0.31 (0.15)
Experimental group	1	0.75	0.99	0.12	0.10
	2	0.89	1.00	0.17	0.18
	3	0.74	1.02	0.26	0.16
	4	0.52	0.75	0.27	0.23
	5	0.47	0.63	0.41	0.28
	6	0.68	0.92	0.42	0.32
	7	0.26	0.26	0.46	0.37
	8	0.68	0.66	0.22	0.22
	9	0.23	0.19	0.39	0.26
	Mean (SD)	0.58 (0.23)	0.71 (0.31)	0.30 (0.12)	0.24 (0.08)

### Association between neurophysiological measures and gait recovery

Correlations between the changes in EEG-EEG pairs exhibiting intergroup differences and gait parameters are shown in Fig. 2b. The temporal asymmetry ratio changes were negatively correlated with the EEG-EEG connectivity changes in the CF-CC, CF-MC, MF-MC, MF-IC, IF-MC, IF-IC, IF-CCP, CFC-CCP, CFC-MCP, MFC-MCP, IFC-ICP, and MC-ICP pairs (ranging from − 0.472 to − 0.561, *p* < 0.05). The changes in gait speed, however, did not correlate significantly to the changes in any EEG-EEG pairs.

Correlations between changes in EEG-EMG pairs exhibiting intergroup differences and gait parameters are shown in Figs. 3c and 4c. For the gamma band, the temporal asymmetry ratio changes were negatively correlated with the EEG-EMG connectivity changes in all pairs (ranging from − 0.472 to − 0.653, *p* < 0.05). For the alpha band, the temporal asymmetry ratio changes were negatively correlated with the EEG-EMG connectivity changes over the middle frontal area (Fz, F2), ipsilesional frontal area (F4, F6) and ipsilesional central area (C4) (ranging from − 0.516 to − 0.643, *p* < 0.05). The changes in gait speed did not correlate with the changes in any EEG-EMG pairs for the alpha or gamma band.

## Discussion

In this study, we demonstrated that EEG-EEG connectivity and EEG-EMG connectivity during walking can be enhanced more by a novel gait training program that uses a turning-based treadmill instead of a regular treadmill. Moreover, the improvement in gait symmetry, but not the gait speed, correlated with the modulations in the EEG-EEG and EEG-EMG connectivity over frontal-central-parietal areas of the brain.

Restoring walking ability, regardless of speed or symmetry, is an important goal of poststroke rehabilitation, but conventional approaches are not always successful at improving gait asymmetry [24, 25]. In our previous and present study, we found that changes in walking speed and temporal symmetry were considered clinically significant as well as statistically significant following the turning-based treadmill training [2, 22]. Interlimb asymmetry of the single limb support time stems from less time spent on the affected limb or more time spent on the unaffected limb. Less time on the affected limb may indicate poorer balance control of the affected limb, and more time on the unaffected limb may be due to difficulty advancing the affected leg [23]. During turning-based treadmill training, the outer limb requires relatively greater activation of the ankle dorsiflexors to swing and greater activation of the ankle plantar flexors for propulsion [26]. Alternately, the inner limb increases extensor muscle activity for stance and increases ankle dorsiflexors use for rapid swing [26, 27]. When we reviewed the individual data on the single support time of the unaffected limb and affected limb, we found that normalization of temporal asymmetry in our participants was primarily due to a reduction in the single support time on the unaffected leg. Therefore, our turning-based treadmill training seems to have a stronger effect on advancing the affected leg than regular treadmill training. In addition, temporal gait symmetry is an important gait parameter that provides information on energy consumption [19, 28]. The gait asymmetry was not improved after 12 sessions of regular treadmill training, which may have be due to task repetition reinforcement of asymmetry on the treadmill [29]. However, the gait asymmetry was improved after turning-based treadmill training in the present study. We thus suggest that this novel training can be applied to improve affected leg advancement and possibly reduce energy consumption in stroke rehabilitation.

In this study, we further demonstrated the improvements in walking speed and gait symmetry accompanied by concurrent modulation in EEG-EEG and EEG-EMG connectivity. Moreover, the larger the changes in functional connectivity were, either in EEG-EEG or EEG-EMG connectivity, the better the recovery of gait symmetry. Therefore, such neuromuscular modulation or neural plasticity induced by training may, at least partially, explain the gait improvement. A previous study suggested that individuals with chronic stroke preserve the capacity to increase synchronization of neural activity between different brain regions as measured by EEG connectivity through neurofeedback training [1]. It has been noted that a challenging, unfamiliar and more difficult task for stroke patients may increase the possibilities for brain activation to stimulate behavioral recovery [3]. Turning-based walking requires more balance and limb coordination than walking in straight line [30]. Therefore, walking on a turning-based treadmill seems to fulfill the features necessary for a task to induce brain activation and thus behavior recovery.

In this study, the EEG-EEG connectivity and EEG-EMG connectivity measured during treadmill walking was used to explore the possible mechanisms for reorganization of functional cortical network and functional coupling between cortical commands and subsequent muscle activation. Involvement of the motor cortex and corticospinal tract in the control of walking has been demonstrated in healthy subjects [5, 8]. However, how novel gait training shapes brain activities and the descending pathway for chronic stroke survivors remains unclear. We revealed both cortico-cortical and cortico-muscular reorganization in the gamma band over the middle central area, middle frontal area, ipsilesional frontal, and contralesional central area can be further enhanced by challenging gait training, such as the turning-based treadmill training in this study. One possible role of coherent oscillation is to link and promote synchronous neural firing between different neuronal populations with different spatial distributions but that are functionally related [31]. The coupling between EEG and EMG indicated that cortical control drives peripheral muscular activities through the corticospinal tract during walking. Increased connectivity in the gamma band after specific walking training is in line with previous results that showed that the peak EEG-EMG coherence frequency always shifted to higher frequency (25–40 Hz) from the beta-band during walking compared to those during static contraction [8]. The gamma-band oscillations in the frontal-central areas play an important role in the execution of the complex goal-directed task which involved motor coordination, cognitive processes and sensorimotor integration [32]. Therefore, the turning-based treadmill training, which includes specific training and requires complex integration of coordinated muscle activity and multiple sensory systems by the cortex, could result in better walking performance.

Assessment of EEG functional connectivity has been widely used to understand the correlation of brain activities in different cortical areas [14]. The coherent neural oscillation in the beta band over the ipsilesional motor cortex was able to predict motor improvements in the first week after stroke [33]. Loss of connectivity thus suggests a low capacity to integrate sensorimotor communication between distant brain regions in stroke survivors [34]. Following 28 days of upper-extremity treatment increased the resting-state connectivity of the beta-band between the ipsilesional primary motor cortex and premotor cortex paralleling the motor gains [34]. A recent study showed that 40-session gait training with powered exoskeletons could improve the strength of functional connectivity in the affected hemisphere [35]. Consistent with above results, we provide evidence that this 12-session turning-based treadmill training for chronic stroke patients could induce synchronization of the cortical network around distinct brain areas together with gait improvement. Furthermore, this challenging turning-based training was demonstrated to be more effective than regular treadmill training with regard to brain modulation and gait performance.

Besides the involvement of neural relocation in the ipsilesional hemisphere, we observed the network contribution over the contralesional frontal-central-parietal area and between the contralesional central area and the lower-extremity muscle for gait recovery. The role of the contralesional motor area for treatment-induced poststroke brain reorganization is controversial. Decreased frontoparietal networking in the contralesional hemisphere has only been found in stroke patients who gained improved motor behavior after ankle robotics training [36]. However, as some recent studies emphasized the role of the contralesional hemisphere during recover [34], our results showed that activation in the contralesional hemisphere did not necessarily contribute to unfavorable recovery, especially for chronic stroke survivors. In addition, motor task selection and duration of training session may also account for such discrepancies.

In addition to the intrahemispheric interactions, increased interhemispheric connectivity after turning-based treadmill training was observed. To our knowledge, evidence of the interhemispheric connectivity induced by lower-extremity training is nonexistent. However, these findings were consistent with previous studies’ findings of the strong influence of the ipsilesional dorsal premotor cortex on its contralesional homologue, with improvements in behavioral performance after 3 weeks of upper limb rehabilitation therapy in stroke patients [37]. Pellegrino et al. [38] also demonstrated that the 12-week robotic treatment for chronic stroke patients improved hand control and modulated the interhemispheric connectivity in the high beta and gamma bands at rest and such changes correlated with hand control improvements. We reported the first evidence that interhemispheric connectivity during walking can be shaped by novel treadmill training. Considering the bilateral involvement of the lower-extremities during walking, neurophysiological mechanisms of walking regarding the interaction between the lesioned and contralesioned hemispheres in stroke patients warrants further study.

Interestingly, increased coherence in the alpha band was only present in the EEG-EMG connectivity in this study. It is well known that the coherence in the 8–12 Hz frequency over the motor area is most prominent during rest, and such coherence is suppressed (or called desynchronization) during voluntary movement [39]. However, a recent study found the coherence between EEG and EMG of TA around this frequency band could be induced by peripheral nerve stimulation while performing static muscle contractions [40]. Petersen et al. also reported a coherence peak at approximately 10 Hz during walking in healthy human subjects [8]. Our present findings may reflect the possibility of treatment-induced reorganization from afferent inputs to the cortical network during walking [2].

It is noteworthy that enhancements in gait symmetry, but not the gait speed, were related to the modulations in EEG-EEG and EEG-EMG connectivity over the frontal-central-parietal areas in the brain. The network changes may thus account for the associated gait recovery underlying the central and peripheral neuromuscular mechanisms. The temporal gait symmetry provides information on the differences of single support times between the 2 legs [20]. As mentioned above, improvement of temporal gait symmetry may be explained by the successful combination of a forced compelled weight-bearing approach and intensive practice of the normal swing phase component. The control of a normal gait pattern demands that patients achieve more muscular control and whole-body coordination [19]. Therefore, the restoration of gait pattern, rather than gait speed, is more likely to relate to the alteration of brain activities. Although speed is the most widely used measurement and can differentiate levels of gait disability, it is determined by multiple factors [20]. Investigation of cortical contribution for gait speed in response to therapeutic approaches is needed in the future. Recently, numerous studies have started to design the EEG-based brain-machine interfaces (BMI) to decode the association between the angles of specific joints and neuroelectric cortical activity during gait training [41, 42]. Since gait symmetry assists in understanding the underlying impairments and treatment-induced cortical changes, a measure of temporal gait symmetry should be included in quantitative gait analysis to better reflect this aspect in gait training with an EEG-based BMI.

This study has several limitations. The sample size is relatively small, and we only assessed limited gait parameters. A larger, randomized controlled clinical trial including comprehensive assessment of gait parameters (i.e., distinct aspects of symmetry or accelerometry signals in time, frequency and time-frequency domains [43]) is needed to validate the reported brain characteristics of the treadmill training in this study. The improved gait speed and temporal symmetry maintained at 1-month follow-up according to our previous works [2], however, whether the brain network-related gait recovery can be seen at follow-up is not known. Additionally, only ambulatory patients with chronic stroke were recruited. Therefore, our results may not extend to individuals with acute stroke or more severe motor deficits. In this study, we only provided 32-channel cortical data and one muscle signal to explore possible connectivity during walking. Future studies should use a combination of anatomical and functional neuroimaging techniques to determine the spatial patterns of brain reorganization after gait training. In addition, the task chosen for neurophysiological assessment was walking on the regular treadmill for both groups. Therefore, the assessed task might be more familiar to participants in the regular treadmill training group, but not to participants in the turning-based training group. Whether neural connectivity is influenced by task familiarity is not clear and needs further elucidation. The lack of a complete understanding of the cortical contribution to walking ability and walking recovery after a stroke is due to lacking of valid tools to thoroughly investigate the functional role of the cortex during walking in humans. As mentioned in the methods section, although we have done our best to exclude all motion artifacts and provide stable task-related EEG signals, we believe future studies using more sophisticated engineering solutions for eliciting artifacts could advance our understanding of the “real-time” brain activities during walking and boost development of effective intervention with a neural basis [44, 45].

## Conclusions

Despite thorough documentation of the unique neural correlations during motor function, previous EEG studies have rarely focused on changes in brain activity during walking in people with stroke after neurorehabilitation. In the present study, we were the first to apply TFCMI, a nonlinear, noise-resistant method within task-related frequency, to explore the EEG-EEG/EEG-EMG connectivity for treatment-induced brain reorganization after a specific gait training program. Our results demonstrated that 12 sessions of this 30-min novel gait training on a turning-based treadmill improved network-related gait recovery with underlying cortico-cortical and corticomuscular mechanisms.

## Abbreviations

BMI: Brain-machine interfaces
CC: Contralesional central area
CCP: Contralesional central-parietal area
CF: Contralesional frontal area
CFC: Contralesional frontal-central area
HEOG: Horizontal electrooculography
IC: Ipsilesional central area
ICA: Independent component analysis
ICP: Ipsilesional central-parietal area
IF: Ipsilesional frontal area
IFC: Ipsilesional frontal-central area
MC: Middle central area
MCP: Middle central-parietal area
MF: Middle frontal area
MFC: Middle frontal-central area
MMSE: Mini-mental state examination
MP: Middle parietal area
pdf: Probability density function
TA: Tibialis anterior
TFCMI: Time-frequency cross mutual information
VEOG: Vertical electrooculography
